# Skin biopsy: an emerging tool for the diagnosis of protein misfolding diseases of the central nervous system

**DOI:** 10.1186/s13024-025-00871-8

**Published:** 2025-07-03

**Authors:** Wei Zhu, Hao-Lun Sun, Yan-Jiang Wang, Xia Lei, Xian-Le Bu

**Affiliations:** 1 Department of Dermatology, Daping Hospital, Third Military Medical University, Chongqing, 400042 China; 2 Department of Neurology and Centre for Clinical Neuroscience, Daping Hospital, Third Military Medical University, Chongqing, 400042 China; 3 Chongqing Key Laboratory of Ageing and Brain Diseases, Chongqing, 400042 China; 4 Institute of Brain and Intelligence, Third Military Medical University, Chongqing, 400038 China; 5 State Key Laboratory of Trauma and Chemical Poisoning (Third Military Medical University), Chongqing, 400042 China; 6 Key Laboratory of Geriatric Cardiovascular and Cerebrovascular Disease, Third Military Medical University, Ministry of Education of China, Chongqing, China

**Keywords:** Neurodegenerative diseases, Protein misfolding, Skin biopsy, Αlpha-synuclein, Tau, Prion, Diagnosis

Abnormal aggregation of misfolded proteins is a common characteristic of many neurodegenerative diseases [1]. Brain protein aggregates formed by phosphorylated tau, amyloid-beta (Aβ), α-synuclein, or prion proteins are toxic and can cause gradual neuronal loss and death in the central nervous system (CNS). These aggregates are linked to many neurodegenerative diseases such as Alzheimer’s disease (AD), and Parkinson’s disease (PD). Rapid advancements in brain PET imaging and body fluid biomarkers have enabled the precise diagnosis of these disorders. However, brain PET scans are expensive, cerebrospinal fluid (CSF) collection is an invasive procedure, and precise diagnostic tools remain limited. Therefore, it is imperative to develop simpler and more accessible diagnostic approaches. Skin tissue shares a common developmental origin with the CNS, as both are derived from the ectoderm during embryogenesis, and skin is rich in nerve fibers. Multiple misfolded proteins, including α-synuclein, tau, and Aβ, are deposited not only in the brain but also in skin tissue [2–4]. The biological classification of PD emphasizes the necessity of detecting pathological α-synuclein for diagnosis, with skin biomarkers being particularly noted as an accessible and promising detection method [5, 6]. Skin biopsy samples are more accessible than brain PET and CSF samples. Therefore, skin biopsy holds potential as a biomarker-based method for the diagnosis of protein aggregation diseases.

Synucleinopathies are defined as a group of neurodegenerative disorders characterized by the abnormal accumulation of α-synuclein in the brain. These include PD, dementia with Lewy bodies (DLB), multiple system atrophy (MSA), and pure autonomic failure (PAF). Recently, Gibbons et al. conducted a blinded, multicenter, cross-sectional study to evaluate the rate of positivity for cutaneous α-synuclein deposition among patients with synucleinopathies (PD, DLB, MSA, and PAF) [7]. The researchers enrolled 151 controls and 277 patients with synucleinopathies diagnosed on the basis of clinical consensus criteria and the findings were confirmed by an expert review panel. Among the participants, 343 participants (223 clinically diagnosed with a specific synucleinopathy and 120 who met the criteria for serving as controls without a synucleinopathy) were included in the primary analysis. Skin biopsy samples were taken from the distal leg, the distal thigh, and the posterior cervical region. Among those patients clinically diagnosed with a specific synucleinopathy, 95.5% (213 of 223) had a skin biopsy sample that was positive for phosphorylated α-synuclein. Specifically, phosphorylated α-synuclein positivity in skin biopsy samples was observed for 92.7% of PD patients, 98.2% of MSA patients, 96% of DLB patients, and 100% of PAF patients. In contrast, only 3.3% (4 of 120) of those in the control group had skin biopsy samples that were positive for phosphorylated α-synuclein. In the secondary analysis, among 27 patients with undifferentiated synucleinopathy (those who did not meet expert panel diagnostic criteria for a specific synucleinopathy), 85.2% (23 of 27) had a skin biopsy sample that was positive for phosphorylated α-synuclein. Among those 58 participants with an unknown diagnosis who did not meet prespecified diagnostic criteria for a synucleinopathy (including 30 patients originally diagnosed with a synucleinopathy and 28 controls), 55.2% (32 of 58) had a skin biopsy sample that was positive for phosphorylated α-synuclein. Moreover, total phosphorylated α-synuclein deposition in the skin was positively correlated with disease severity.

Tauopathies are a group of neurodegenerative disorders characterized by the abnormal deposition of hyperphosphorylated tau protein in the brain; these disorders include AD, Pick’s disease (PiD), progressive supranuclear palsy (PSP), and corticobasal degeneration (CBD) [8]. Wang et al. assessed the diagnostic potential of tau seeding activity in the skin of participants with neuropathologically confirmed tauopathies. They enrolled 46 AD, 5 CBD, 33 PSP, and 6 PiD patients and 43 healthy and examined pathological tau seed amplification assay (tau-SAA) by performing ultrasensitive real-time quaking-induced conversion (RT-QuIC) in skin biopsy samples taken from the lateral region to C7. They reported that skin tau-SAA had a higher sensitivity (75–80%) and specificity (95–100%) for detecting brain tauopathy. Skin tau-SAA could differentiate AD from non-AD tauopathies with a sensitivity of 80.49% and a specificity of 95.35%. In addition, skin tau-SAA findings were associated with Braak staging in autopsied brain tissues, indicating that skin tau seeding activity can reflect the severity of tauopathies.

Human prion diseases are fatal neurodegenerative disorders characterized by the accumulation of misfolded prion protein in the brain. Chen et al. compared the diagnostic efficacy of CSF and multisite skin biopsies in vivo to evaluate the potential of skin RT-QuIC technology in the diagnosis of prion diseases [9]. For this study, the researchers enrolled 101 patients with prion diseases (including both sporadic and hereditary types) and 23 non-prion disease patients. Skin biopsy samples were collected from the following sites: the area near the ear, upper arm, lower back, and inner thigh. The analysis revealed that the RT-QuIC sensitivity across different skin sites was 84.9% for the area near the ear, 80.0% for the upper arm, 83.7% for the lower back, and 84.8% for the inner thigh under three dilution conditions. Among these areas, the area near the ear presented the greatest consistency under various dilution conditions. When any two samples from the area near the ear, the inner thigh, and the lower back were combined, the RT-QuIC sensitivity reached 92.1% (93 of 101), which was significantly greater than that of CSF analysis alone (75.5%). Furthermore, when samples from all the skin sites were combined, the sensitivity further increased to 95.0%. This study highlights that the prion seeding activity determined by RT-QuIC in skin biopsy samples is superior to that determined by CSF analysis for the diagnosis of prion diseases.

The above studies demonstrated that a high proportion of individuals with synucleinopathies, tauopathies and prion diseases had abnormal accumulation of α-synuclein, tau, or prion, respectively, in the skin, and quantitative measurements of these misfolded proteins in the skin could serve as diagnostic biomarkers for these neurodegenerative disorders (Fig. 1). Although 95.5% of patients clinically diagnosed with specific synucleinopathies had skin biopsy samples that were positive for phosphorylated α-synuclein, the detection rate was lower for PD patients than for MSA, DLB and PAF patients. These findings indicate that PD, DLB, and MSA exhibit differences in skin synuclein deposition patterns, which may reflect distinct pathological mechanisms and clinical phenotypes. Skin tau seeding activity exhibited moderate diagnostic sensitivity but high specificity for tauopathies. Although the diagnostic accuracy of skin tau seeding activity for AD is less than that of blood biomarkers such as phosphorylated tau 231 and 217 [10], skin tau seeding activity may serve as a supplementary tool for the diagnosis and differential diagnosis of tauopathies. Currently, the diagnosis of prion diseases in clinical practice is often challenging. Prion seeding activity as determined by RT-QuIC analysis of multisite skin biopsy samples was significantly more accurate than CSF activity for the diagnosis of prion diseases, and RT-QuIC analysis may serve as a convenient method for the early and accurate diagnosis of prion diseases.

**Fig. 1:**
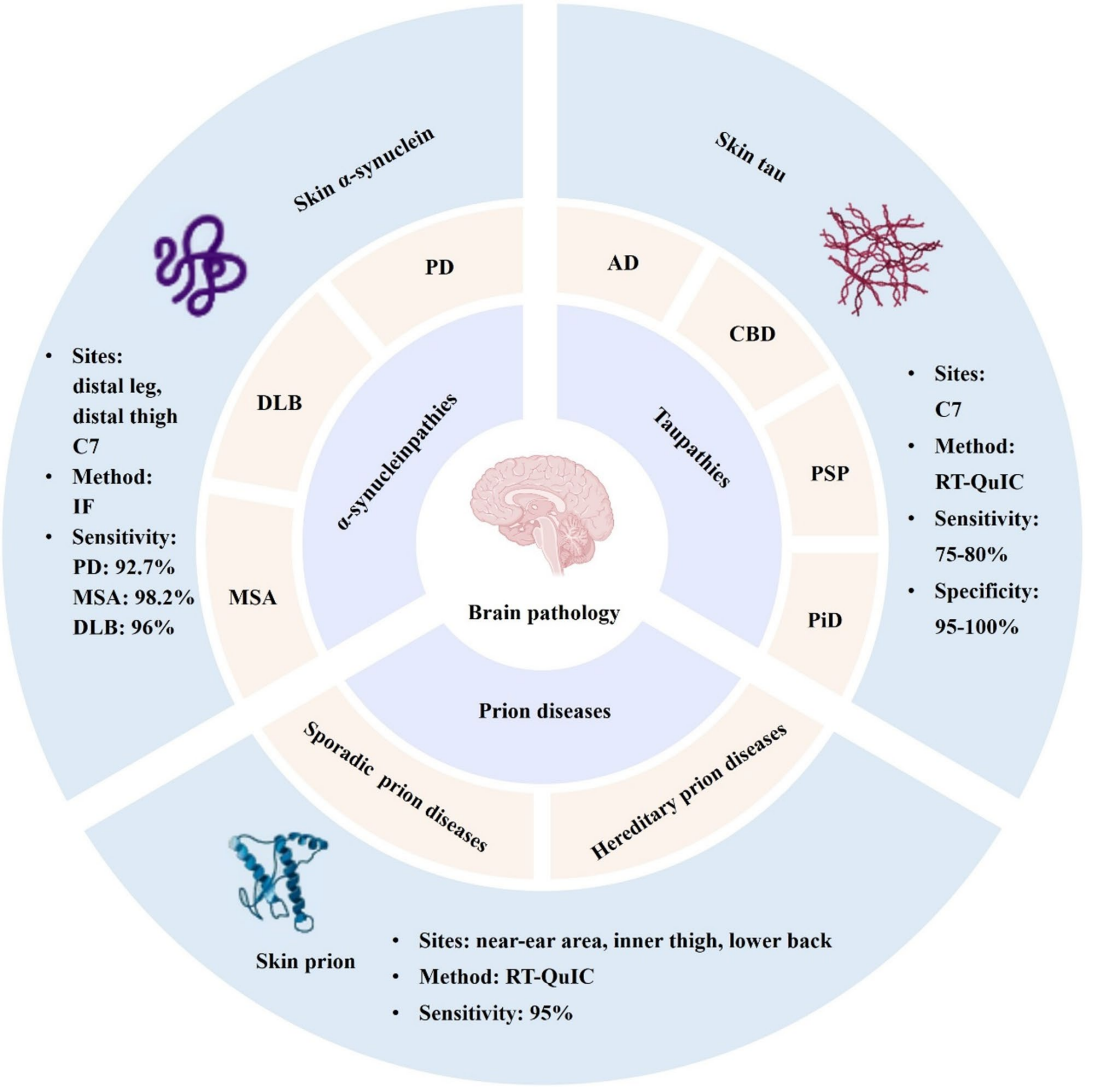
Skin-based biomarkers for the diagnosis of protein misfolding diseases of the central nervous system. Multiple misfolded proteins, including α-synuclein, tau, and prion, are deposited not only in the brain but also in skin tissue. The detection of pathological protein aggregates in skin biopsy samples holds significant promise for the diagnosis of neurodegenerative diseases. Abbreviations: AD, Alzheimer’s disease; C7, seventh cervical vertebrum; CBD, corticobasal degeneration; DLB, dementia with Lewy bodies; MSA, multiple system atrophy; PD, Parkinson’s disease; PiD, Pick’s disease; PSP, progressive supranuclear palsy; IF, immunofluorescence; RT-QuIC, real-time quaking-induced conversion

There are several limitations in these studies. First, the diagnosis of synucleinopathies was dependent on clinical symptoms and evaluation by an independent expert review panel, and direct evidence of brain α-synuclein pathology was lacking. Second, the study of skin tau seeding activity for diagnosing tauopathies was limited by small sample sizes, and further research is needed to determine whether combining multisite skin biopsy sample analysis of tau seeding activity could increase the diagnostic efficacy of this method for tauopathy diagnosis.

Skin-based biomarker analysis for the diagnosis of neurodegenerative diseases has the following unique advantages: skin samples are easily obtained via biopsy, and this detection method costs less than brain PET; skin biopsies are less invasive and better tolerated than lumbar punctures are; and skin biopsies are convenient for dynamically monitoring the disease progression of patients during follow-up. However, several issues need to be addressed before skin biopsies for neurodegenerative disease diagnosis can be widely implemented in clinical practice. First, the primary deposition sites of pathogenic proteins in the skin may differ among various neurodegenerative diseases, highlighting the need to identify the most diagnostically effective skin biopsy locations with the highest diagnostic efficacy. Second, the temporal relationship between pathogenic protein deposition in the skin and the brain must be clarified to evaluate the potential of skin biopsies for early diagnosis. Third, the correlation between abnormal protein aggregates in the skin and disease-related biomarkers in CSF and the brain, as well as the association of skin protein aggregation with disease severity, should be investigated to determine the utility of this method for predicting disease progression and prognosis.

In conclusion, the detection of pathological protein aggregates in skin biopsy samples holds significant promise as a diagnostic biomarker of neurodegenerative diseases. Large-scale cohort studies are needed to validate the clinical utility of skin-based diagnostic approaches for these disorders in real-world settings.

## Data Availability

No datasets were generated or analysed during the current study.

## Abbreviations

AD: Alzheimer’s disease
Aβ: Amyloid-beta
CBD: Corticobasal degeneration
CNS: Central nervous system
CSF: Cerebrospinal fluid
C7: Seventh cervical vertebrum
DLB: Dementia with Lewy bodies
MSA: Multiple system atrophy
PAF: Pure autonomic failure
PD: Parkinson’s disease
PiD: Pick’s disease
PSP: Progressive supranuclear palsy
RT-QuIC: Real-time quaking-induced conversion
SAA: Seed amplification assay
